# Are Myths and Preconceptions Preventing Us from Applying Ionic Liquid Forms of Antiviral Medicines to the Current Health Crisis?

**DOI:** 10.3390/ijms21176002

**Published:** 2020-08-20

**Authors:** Julia L. Shamshina, Robin D. Rogers

**Affiliations:** 525 Solutions, Inc., P. O. Box 2206, Tuscaloosa, AL 35403, USA

**Keywords:** ionic liquids of active pharmaceutical ingredients (API-ILs), antiviral API-ILs, solubility, polymorphism, cocrystals, pharmaceutical salts, acyclovir, GSK2838232

## Abstract

At the moment, there are no U.S. Food and Drug Administration (U.S. FDA)-approved drugs for the treatment of COVID-19, although several antiviral drugs are available for repurposing. Many of these drugs suffer from polymorphic transformations with changes in the drug’s safety and efficacy; many are poorly soluble, poorly bioavailable drugs. Current tools to reformulate antiviral APIs into safer and more bioavailable forms include pharmaceutical salts and cocrystals, even though it is difficult to classify solid forms into these regulatory-wise mutually exclusive categories. Pure liquid salt forms of APIs, ionic liquids that incorporate APIs into their structures (API-ILs) present all the advantages that salt forms provide from a pharmaceutical standpoint, without being subject to solid-state matter problems. In this perspective article, the myths and the most voiced concerns holding back implementation of API-ILs are examined, and two case studies of API-ILs antivirals (the amphoteric acyclovir and GSK2838232) are presented in detail, with a focus on drug property improvement. We advocate that the industry should consider the advantages of API-ILs which could be the genesis of disruptive innovation and believe that in order for the industry to grow and develop, the industry should be comfortable with a certain element of risk because progress often only comes from trying something different.

## 1. Difficulties with Current Pharmaceuticals

**Figure 1:**
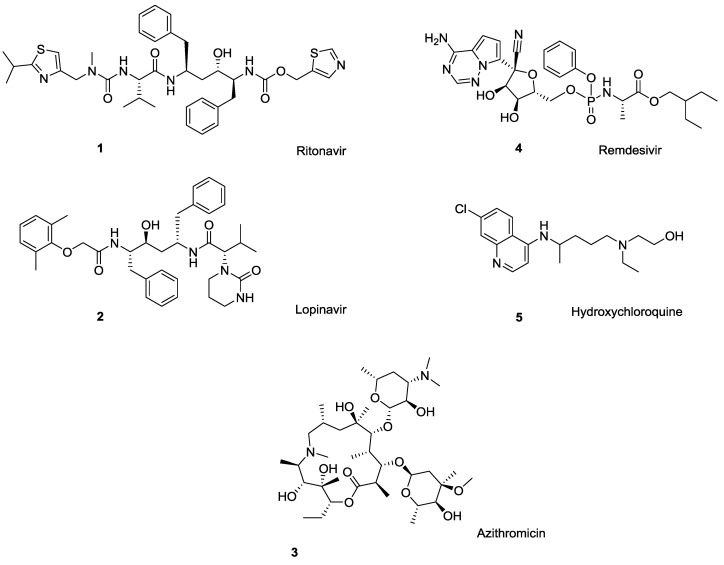
Drugs currently under clinical trials for COVID-19 treatment: ritonavir (**1**), lopinavir (**2**), azithromycin (**3**), remdesivir (**4**), and the anti-malaria drug hydroxychloroquine (**5**). All bear ionizable functionalities.

The ongoing COVID-19 situation has demonstrated how critical it is to have antiviral treatments available with little warning. At the moment, there are no U.S. Food and Drug Administration (U.S. FDA)-approved drugs for the treatment of COVID-19, although several antiviral drugs are available through an U.S. FDA emergency use authorization (EUA), and are under clinical trials [1]. These include antiviral medications such as ritonavir/lopinavir combination, azithromycin, remdesivir, tocilizumab, sarilumab, leronlimab, and the anti-malaria drug hydroxychloroquine (Figure 1) [2,3]. A closer look at this list of medications shows that more than half of these drugs can undergo polymorphic transformation (i.e., crystallize in different solid state forms), resulting in changes in physical and chemical properties that can significantly impact the drug’s solubility, water sorption and desorption capacity, stability, etc., [4] and, therefore, affect the drug’s safety and efficacy.

A classic example of polymorphic conversion that negates the efficacy of a drug is ritonavir (Norvir^®^), an antiretroviral drug used to treat HIV/AIDS. Development costs of ritonavir [5] exceeded $200M [4], but the drug was found to suffer from polymorphism and subsequent changes in bioavailability *after successful product launch* [6,7,8,9]. This required market withdrawal and subsequent re-formulation [10]. Among other drugs currently investigated for COVID-19 treatment, HIV protease inhibitor lopinavir exists in as many as ten polymorphic forms [11] the macrolide antibiotic azithromycin (Zithromax^®^) occurs as three polymorphs [12,13], and a final form of the malaria medication hydroxychloroquine monosulfate is reported to exist as two polymorphs [Among other antivirals (i.e., not considered for COVID-19 treatment), the guanine nucleoside ganciclovir [14,15], a commonly used antiviral for herpes simplex virus acyclovir [16], and the anti-hepatitis drug clevudine [17] are polymorphic.

In fact, more than 50% of *all* active pharmaceutical ingredients (APIs) suffer from polymorphism [18,19,20], defined in U.S. FDA guidelines as a phenomenon that “can affect the quality, safety, and efficacy of the drug product” [21]. Since predicting the likelihood of polymorphism in a specific molecule presents a significant challenge, laborious screening for polymorphism of all accessible solid-state forms, including hydrates and solvates, has been added to the drug development stages [22,23].

Making matters worse, ca. 40% of currently marketed drugs and 80% of pipeline candidates meet the Biopharmaceutical Classification System’s (BCS) definition of poorly soluble (classes II and IV) drugs [24,25], which can result in halting drug candidate development because of difficulties with achieving the needed drug concentration in systemic circulation for desired pharmacological response. Examples of poorly bioavailable antivirals include a neuraminidase inhibitor zanamivir, active against a broad panel of influenza A and B strains [26], a selective inhibitor of the replication of herpes simplex virus (types 1 and 2) acyclovir [27], nucleosides tenofovir and adefovir, etc., [28]. The decreased solubility that can also arise from a particular polymorphic form interferes with drug screening results by causing miscalculated bioactivity, reduced high throughput screening-hit rates, data discrepancies, erroneous structure activity relationships data, etc., [29]. As such, identifying opportunities and a rationale for “reformulation” of anti-viral drugs into more soluble and bioavailable form is a subject of high importance.

## 2. Current Tools to Reformulate APIs into More Bioavailable Forms

By far, the most common strategy to overcome solubility and bioavailability problems with a given API is formulation as a salt. More than 50% of all drug molecules [30], and 50% of the top 200 prescription drugs in the U.S., are used as salts [31]. Simply pairing of an ionizable drug with a pharmaceutically acceptable counterion to form a neutral salt is utilized by both “big pharma” and smaller pharmaceutical companies who deal with patent expiration issues in order to develop a generic alternative. In many cases, the salt form of an API can address both solubility and bioavailability issues, as a result of increased bioavailability, improved absorption, and enhanced in vitro effectiveness.

As an alternative to formulating APIs as salts, the concept of cocrystals was introduced where an ionizable or nonionizable API is paired with an excipient molecule which results in the crystallization of both in a new solid-state form [32,33]. According to the U.S. FDA guidelines, pharmaceutical cocrystals are crystalline materials composed of two or more different molecules, typically an API and cocrystal former, in the same crystal lattice.

Banerjee has shown using saccharinate salts (e.g., quininium, zolpidemium, and amlodipinium saccharinates), that their solubilities were greater than that of the respective cocrystal (e.g., saccharine—piroxicam), because the hydrogen bond of the cocrystal breaks apart as soon as the compound is placed into solvent, and the API’s solubility is not changed from its parent form [34]. Very recently, the question of whether solubility enhancement is better in a salt or cocrystal was reviewed [35], and it was shown that because of differences in the interaction of salts and cocrystal with a solvent, the solubility enhancement was not directly comparable and depends on each API’s characteristics.

The first regulatory classification that addressed both cocrystals and pharmaceutical salts was issued in 2013 [36] and was later replaced with a revised final guideline in 2018 [37]. In this document, the U.S. FDA finalized the regulatory approach and included materials, that the U.S. FDA “has not previously evaluated and determined to be pharmaceutical cocrystals” [37]. New regulatory classification placed cocrystals into the position similar to that of a polymorph of the API, “as a special case of solvates and hydrates, wherein the coformer (. . .) is typically nonvolatile.”

According to U.S. FDA regulations, different cocrystalline forms are considered the same API, drug product intermediates, or as in-process materials [37]. Key statements within the U.S. FDA designation are the components that interacted non-ionically in a pharmaceutical cocrystal, and that the components are present in a defined stoichiometric ratio within the crystal lattice. Thus, cocrystals represent a tool to impart certain properties into an existing API. For drug applications containing cocrystals, an in vitro evaluation based on dissolution and the proof that the API dissociates from its cocrystal former before reaching the site of action is generally considered sufficient. Contrarily, in pharmaceutical salts the components might have different stoichiometries and must interact ionically; thus different salt forms of the same active moiety are considered to be different APIs by regulations [37]. Thus, even though a strong consensus on the need to define cocrystals more broadly and to classify them similar to pharmaceutical salts was reached in 2012 [38], and the difference between pharmaceutical salts and cocrystals lies only in the extent of proton transfer which is somewhat arbitrary [39], there is a strict separation between salt and cocrystal from a regulatory standpoint.

Despite a potentially harder regulatory pathway, preparation of salts from poorly water-soluble drugs would seem to be the most effective approach to improve the pharmacokinetic profile of a drug and manage solubility and dissolution rate issues. Preparation of salts is a part of a broad salt selection program during drug development where the salt form most suitable for development is identified and counterion selection is an important and critical step of this program [40]. The initial stages of salt selection (Tiers 1/2) evaluate drug crystallinity by means of high throughput screening [41], and a high degree of crystallinity is by far the most essential criteria for the selection of a particular salt form. Immediately after crystallinity evaluation, hygroscopicity screening (Tier 3) is conducted that identifies a drug’s humidity and temperature dependent changes [42]. The final step of the salt selection protocol (Tier 4) is the evaluation of aqueous solubility/bioavailability. Other criteria include chemical and solid-state stability (this includes polymorphic stability), and excipient compatibility. Thus, the number of available counterions for salt formation is rather limited to those that would result in a biocompatible, highly crystalline, non-hygroscopic salt form—typically, chloride, bromide, mesylate, acetate, and fumarate salts are used the most frequently for basic drugs, while sodium, magnesium, and potassium are used the most frequently for acidic drugs [43].

## 3. What about API Ionic Liquids (API-ILs) as Alternative to Common Strategies?

Today the pharmaceutical industries seem focused only on solid crystalline or amorphous pharmaceutical salts. However, in 2007 we challenged this concept [44] and asked why not consider ionic liquids (ILs, liquid salts that melt below 100 °C [45]) which can also incorporate APIs into their structures. If one were to focus on API-ILs that melt below body temperature of 37 °C, one would have *pure liquid salt forms* of APIs allowing for all the advantages that salt forms provide from a pharmaceutical standpoint, but without being subject to problems of the solid state such as polymorphism. API-ILs are easily accessible, and have shown to be effective in the development of novel forms of antimicrobial, fungicidal, and pain-relieving medicines [46,47,48,49,50].

Just like for solid pharmaceutical salts, the IL approach is cost-effective and less risky when compared to new drug development. Through a rational combination of discrete cationic and anionic derivatives of approved APIs, API-ILs allow tuning the biological, chemical, and physical properties of the drugs while maintaining their biological activity, by design. Among the most impressive examples of solubility increase are choline-based API-ILs: Choline sulfasalazine, which demonstrated 4000-fold solubility enhancement compared to sulfasalazine [46]; choline acyclovir, which showed a 450 times solubility improvement [47]; choline nalidixate, the water solubility of which is 5000 times higher than that of the parent drug; and choline niflumate, where water solubility is as much as 56,000 times higher than that of the parent API [48]. We will not go into examples of API-ILs in detail here, as one can find extensive reviews and book chapters on the subject [8,49,50].

API-ILs are applicable to not only oral but also dermal or transdermal delivery [51], providing a tremendous opportunity for developing drugs with tunable permeability through biological barriers. For instance, the API-IL for transdermal drug delivery, lidocainium etodolac [52] that completed Phase III trials [53,54] showed higher biological activity than either drug alone. API-ILs provide maximal dosing flexibility and minimum dose preparation errors because of being liquids (and thus measured by volume), eliminate risk of aspiration or choking, and allow use of a single formulation over a wide range of ages including neonates or the elderly. API-ILs can customize the hydrophilicity or hydrophobicity of the targeted APIs. Developing new API-ILs can be considered for drugs that were never commercialized for their originally developed targets resulting in “new old” pharmaceuticals with improved pharmacokinetic profile which could be crucial in combating forthcoming threats. Yet, even though API-ILs represent an enabling technology platform that might serve a global pharma market with integrated drug design, development, and manufacturing, there are countless “proof-of-concept” studies with only limited examples of commercialization efforts.

## 4. The Myths and Perceived Concerns Holding Back Implementation of API-ILs

Before we debate the concerns with API-ILs, we would like to address the question of common generalities that follow the IL field [55] and emphasize the fact that because ILs are such a broad class of compounds, there is no single common characteristic that can describe them all, other than the definition as a low melting salt. But because of generalizing a single property of a single IL to every IL, a mythology builds up, such as ILs are green or ILs are toxic [56,57], which prevents rational thought into the properties of an IL based on its chemical composition. This also often leads to researchers using the wrong salt for a given application, simply because no thought went into the chemical identity and properties of the component ions. Thus, the “problems” associated with using the API-IL strategy do not come from the fact that they are ill-suited for use as pharmaceuticals, but from the fact that people think they are. Here we will examine some of the most voiced concerns about using an IL form of an API.

### 4.1. Physical Properties: Viscosity, Hygroscopicity, and Stability

An often-expressed concern in using API-ILs is that ILs are viscous, and therefore, could cause manufacturing, handling, and dosing challenges [58]. Another concern is that API-ILs are hygroscopic and could impact the stability during manufacture, handling, and storage [58]. Finally, there is a stability concern, where it is assumed that the solid crystalline state is always more stable [59].

A comprehensive summary of viscosity for ILs [60] retrieved over 13,000 data points of temperature- and pressure-dependent viscosity of 1484 ILs from more than 450 research papers, indicated viscosity values from 4.1 to 27,000 mPa•s. The desired viscosity can be achieved by choosing proper combinations of cations and anions, and there are predictive models to calculate density and viscosity (e.g., using quantitative structure property relationships (QSPR) [61]). Some already developed strategies to tune physical properties include formation of double salt ionic liquids (DSILs, ILs with more than one type of cation or anion) which can provide API-ILs with nonlinear, unexpected changes of physicochemical properties compared to the constituent ions [62].

There are multiple examples of imparting greater hydrophilicity or hydrophobicity into targeted API-ILs, which can be miscible, partially miscible, or fully immiscible with water and other polar organic solvents. There are multiple U.S. FDA-approved ions for pharmaceutical salts, use of which would result in highly hydrophobic salts if employed (e.g., Docusate [44], 1,4-Bis(2-ethylhexoxy)-1,4-dioxobutane-2-sulfonate). Tuning the hydrophilic-lipophilic balance for an API-IL could be as simple as proper choice of counterion [63].

For the determination of thermal stability, drug candidates are evaluated according to U.S. FDA guidelines [64], under both accelerated temperature increase and under long-term storage conditions. There are no definite values of what to consider a thermally stable API. API-ILs can also be quite thermally stable, if the ions are chosen for that purpose. Interested readers can access a review article [65], which summarized thermal stability (expressed as 5% decomposition, T_5%onset_) of many API-ILs. It was shown that the thermal stability depends on both the cation and anion, as expected: out of 40 reviewed API-ILs, 10% had thermal stability of <120 °C, 60% had thermal stability of >190 °C, and for the other 30% the information was not provided. Thermally stable examples included ampicillin-ILs [66] which exhibited thermal decomposition temperatures well above 200 °C (e.g., T_5%onset_ of cetylpyridinium ampicillin is 269.39 °C, choline ampicillin—221.29 °C, and trihexyltetradecylphosphonium ampicillin—297.65 °C) and “sweeteners” [67] whose thermal decomposition temperatures were above 200 °C (e.g., T_5%onset_ of didecyldimethylammonium saccharinate is 214 °C, hexadecylpyridinium acesulfamate—267 °C, and didecyldimethylammonium acesulfamate—232 °C). Among other thermally stable ILs, lidocainium docusate with T_5%onset_ of 222 °C [44] and tetrabutylphosphonium salicylate with T_5%onset_ of 307 °C [68] are worth noting.

### 4.2. Toxicity

Toxicity of API-ILs is often considered an unknown or a risk, typically for lack of data. A recent review [49] lists the poorly understood mechanisms of action of API-ILs including relationships between the API-IL structure and its nano-/microscale organization and the need for more fundamental studies of chemical reactivity of ions in the liquid phase, among other toxicity-related concerns. Others cite insufficient and non-systematic comparative studies on different types of API-ILs which are restricted to studies of the systems of particular types made of mostly two ions [69].

Rapid expansion of interest in API-ILs has brought about many recent discoveries including association of the ions in aqueous solution and at the cell membrane [70], transport studies of the API-ILs that show the formation of ion-pair complexes [71], ion-pairing ion combinations maintaining the drug in its ionized form [72], formation of strongly hydrogen-bonded complexes similar to ion pairing but without formal charges [51], and so on. This does raise a legitimate concern on whether there could be specific synergistic or antagonistic effects on bioavailability and toxicity. In fact, the particular toxicity question lacks appropriate decision-support tools to facilitate making the right calls, therefore, a primary goal now is to develop a flexible toxicity-testing strategy for existing API-ILs, improve various testing approaches, and collect more data.

### 4.3. Difficulties in Characterization

Critics of API-ILs often use the excuse that there are only so many analytical methods for testing API-ILs, and there are no established U.S. FDA guidelines regarding current good manufacturing practices (GMP) that includes manufacturing, performance testing, monitoring, process validation, release and stability testing, packing, storage, etc., for API-ILs [73]. We agree that implementation would require novel standardized methodologies at various scales and new GMP processing requirements to support early development and clinical and/or commercial production. We would see these as additional commercial opportunities and do not believe there is inherently anything about API-ILs that makes them difficult to characterize; they are just new and novel methods and innovative thinking will be required.

It is likely that laboratory characterization techniques will not meet U.S. FDA standards or the standards employed by commercial pharma producers who manufacture pharmaceutical salts; however, the problem is definitely not in the lack of such methods. A wide variety of techniques—including standard ultraviolet–visible (UV-Vis) spectroscopy, nuclear magnetic resonance (NMR) spectroscopy, Fourier-transform infrared (FT-IR) spectroscopy, and high-performance liquid chromatography (HPLC) techniques [74,75,76,77] can be used for API-ILs’ analysis, and many methods for measuring solubility and partition properties can be transferred from other areas. There are multiple analytical methods already established for API-ILs: high-resolution magic angle spinning (HR-MAS) NMR spectroscopy to estimate ion–ion interactions [78,79], pulse-field-gradient (PFG-NMR) [80] techniques to estimate extent of interionic complexation, FT-IR spectroscopy and Walden plots to evaluate ionization [81], dynamic light scattering (DLS) to study ion pairing in solutions [82], etc. But there is still a lack of applicability of standby analytical tools, an absence of clear guidance about when to use a particular technique over another, and the need to generate new tools.

## 5. Does the Pharma Industry Work with Liquids?

Surprisingly, given the pushback against using API-ILs because they are liquid, the pharma industry works with liquids all the time. Acceptable liquid dosage forms (defined as a substance in the fluid state of matter having no fixed shape but a fixed volume) include a continually developing list of new solutions [43], oral liquids (e.g., syrups or pediatric drops) [83], liquid suspensions, emulsions or other colloidal systems (produced by colloid mills and homogenizers) [84,85], injections, and liquid drug formulations (based on eutectic mixtures [86]).

Many of these complex formulations include a variety of compounds in addition to the active, such as solubilizers, stabilizers, surfactants, buffers, tonicity modifiers, bulking agents, viscosity enhancers/reducers, surfactants, chelating agents, and adjuvants, to ensure stability [87,88]. Importantly, for making formulations, one has to take into account the factors that can influence bioavailability such as drug particle size, ability to form polymorphs under certain conditions, pH, and bioavailability due to the presence of additives. Some APIs are extremely difficult to formulate at all, such as water-sensitive APIs [89,90], or poorly soluble APIs [91]. This would suggest that making a formulation of a solid API is not easier, but more complex than making API-ILs.

If this is the case—why does the overwhelming preference for solid APIs arise? Doesn’t the reliance on (some could say requirement of) solid forms of APIs focus on finding a solution to specific, near-term problem, while missing opportunities to address underlying strategic issues? If one still has concerns with dosing problems, API-ILs do not even have to be liquids! Supported ionic liquid phases (SILPs), materials in which API-ILs are noncovalently attached to the silica carrier, facilitate the handling of the APIs [92]. A number of API-ILs (tetrabutylphosphonium ibuprofenate [P_4,4,4,4_][Ibu]; choline acyclovir [Cho][Acy]; *N,N,N*-tributyl-*N*-methylammonium acyclovir [N_1,4,4,4_][Acy]; *N*-hexadecyl-*N,N,N*-trimethylammonium acyclovir [N_1,1,1,16_][Acy]) have been shown to be successfully loaded onto silica [93], and can be released from it in different media when necessary.

## 6. Are API-ILs Arriving in Pharma? The case of Glaxo Smith Kline GSK2838232

**Figure 2:**
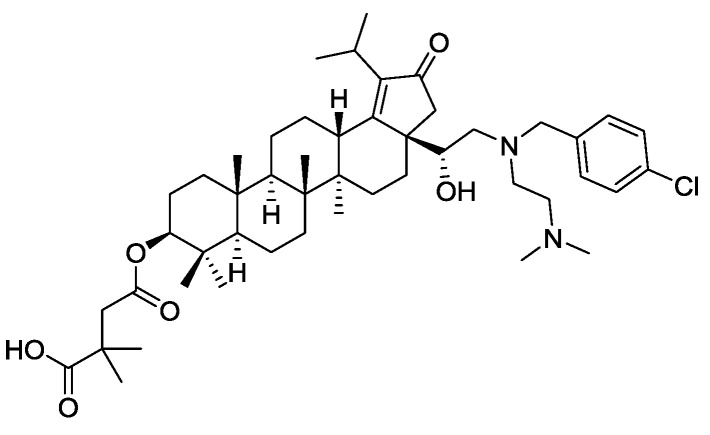
Structure of GSK2838232, a reverse transcriptase inhibitor for the treatment of HIV.

In the COVID-19 world, one wonders why IL forms of antivirals have not been more studied to provide new administration and possibly efficacy routes for known antiviral agents. In fact, anti-viral API-ILs may be arriving—GlaxoSmithKline (GSK) has recently reported a reverse transcriptase inhibitor for the treatment of HIV virus [94] a botulin derivative under code name GSK2838232 [95,96]. The antiviral drug inhibits the activity of a viral DNA polymerase required for replication of HIV retrovirus [97]. The amphoteric compound (see full name in Ref. [98], and a structural formula in Figure 2) possesses two tertiary amines and a carboxylic acid group, being suitable for the conversion into an API-IL through pairing with either acidic or basic counterion. This reverse transcriptase maturation inhibitor was found to be potent against a broad range of clinically relevant sequences of major structural proteins of HIV virus (group-specific antigens or gags). As an API, GSK2838232 successfully passed Phase I clinical studies [99] and is currently in phase II clinical trials [100] for the treatment of HIV infections.

The API, however, presented issues with solubility (<0.01 µg/ mL), where the particle/suspension settling risk could potentially lead to caking, particle growth and/or agglomeration, resuspension challenges, particulate clogging of syringes, etc. Various API suspension formulation variations were tested with all presenting the particle settling issue. In search of long acting injectables from this antiviral, and attracted by new opportunities API-IL could provide, GSK conducted a study of the preparation and testing of GSK2838232-ILs for this purpose [101], and patented the invention of API-ILs [102]. The company noted that while API-ILs “are not prevalent in the pharmaceutical industry and are not used as long-acting injectable formulations,” they “could lead to new pharmaceutical compositions and formulations for the purpose of inhibiting HIV replication and treating HIV patients” [95].

Several long acting formulations of GSK2838232 were compared [103] including aqueous crystalline suspensions (of various particle sizes, versions, and forms), API-ILs, and implantable devices which were 3D printed using poly lactic-co-glycolic acid to achieve various architectures for different drug release profiles. For this, GSK2838232-ILs were prepared by combining the API with several suitable counterions to form liquid salts at room temperature. When GSK2838232 was paired with ascorbic acid, gentisic acid, glucuronic acid, D-gluconic acid, R-(-)-mandelic acid, hippuric acid, shikimic acid, thymine-1-acetic acid, nicotinic acid, cysteine, and sorbic acid in a 1:1 ratio, the resultant products were liquids at room temperature. For two other acids, glutaric and lactic, an “oligomeric approach” [104] was used where an API:acid ratio of 2:1 resulted in liquid API-ILs. All API-ILs exhibited the required stability for use as a long acting injectable product suitable for administration to humans.

Pharmacokinetic evaluations of GSK2838232 using Sprague-Dawley rats were carried out to evaluate each approach [103]. Significantly higher drug concentrations were achieved in API-IL formulations which exceeded the maximum concentrations that could be achieved by a typical solid suspension (150–250 mg/mL). After treatment with API-ILs, almost 50% higher concentration of active in rat plasma (110 ng/mL) was also detected when compared with a simple API suspension (an important benefit for any parenterally administered drug limited by dosing volume). The API-IL formulations also resulted in overall higher drug exposures in rats over the time course which could translate into a less painful experience for patients.

The API-IL strategy is also suitable for using two APIs simultaneously [105] and for GSK, this would suggest two APIs which are active for HIV resulting in a highly concentrated fixed dose combination injection instead of having two separate injections, thus reducing the burden on the patient [102]. The API-ILs could also act as a vehicle (e.g., solvent) for another HIV medication to create a fixed dose combination [102]. Other benefits include improved stability, improved solubility, ability to control release rates, and ease of delivery. GSK’s main concerns involve absence of GMP manufacturing equipment and a potential need of a custom device to accommodate high viscosity [101].

## 7. Other Antiviral API-ILs Already Exist: The Case of Acyclovir

Before the outbreak of COVID-19, Johns Hopkins’ researchers stated that “broad-spectrum [antiviral] therapeutics should be pursued given their potential value” [106]. Today, when the global health crisis in respect to the COVID-19 pandemic is continuing to grow, scientists are working on potential treatments. These treatments include existing antivirals already in use for other illnesses, and that are currently under clinical studies despite aforementioned problems of poor bioavailability. Other drugs never even made it this far being abandoned before clinical studies were conducted. If the current approach to COVID-19 drugs is to repurpose an existing “problematic” API, why not to use antivirals made into API-ILs which can overcome these efficacy problems, in critical situations?

We have already shown that the poorly soluble antiviral drug acyclovir (9-[(2-hydroxyethoxy)methyl]guanine), a nucleoside of the guanosine family can be made into API-ILs [47]. Acyclovir can act as either hydrogen acceptor or hydrogen donor (pK_a Acidic_ 11.98, and pK_a Basic_ 3.02), so one can take advantage of the amphoteric character of acyclovir and convert it into an API-IL as either the anion or the cation [47]. Acyclovir itself is polymorphic, and its low systemic bioavailability (10–20% [107]) is a result of limited aqueous solubility (1.42 mg/mL at 25 °C 107]) and low permeability [108,109,110,111], that requires the drug to be administered in very high doses (200–1000 mg, 3–4 times daily) or intravenously (IV); with increasing doses its oral bioavailability decreases [107].

**Figure 3:**
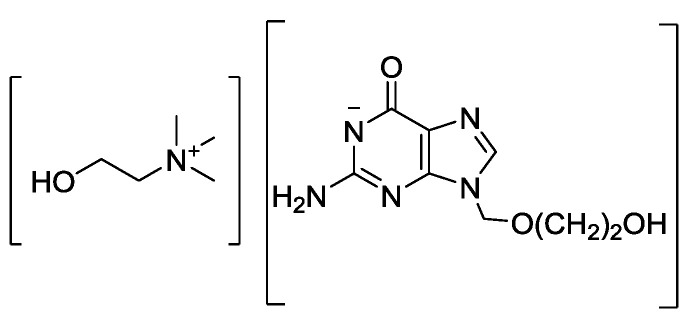
Structure of choline acyclovir [Cho][Acy].

We have shown how the amphoteric acyclovir can form antiviral API-ILs through pairing acyclovir with either cations (choline, phosphoniums, and ammoniums), or anions (chloride or docusate) [47]. All of the prepared salts (two of which, choline acyclovir [Cho][Acy] and acyclovir docusate [H_2_Acy][Doc], adhered definition of API-IL with no melting point (mp) detected, three more, [P_4,4,4,4_][Acy], [N_4,4,4,4_][Acy] and [N_1,1,1,16_][Acy] exhibited melting points below 100 °C [47], satisfying the “classic” definition of ILs [45], and one high-melting point salt acyclovir chloride [H_2_Acy]Cl) demonstrated much higher solubility compared to neutral acyclovir in water and three physiological media (phosphate buffer saline PBS, simulated gastric fluid SGF, and simulated intestinal fluid SIF). The solubilities of [Cho][Acy] (Figure 3) were the highest in water (~600 mg active/mL), showing ca. 450 times solubility enhancement compared to neutral acyclovir (600.0 mg active/mL for [Cho][Acy] vs. 1.49 mg active/mL for acyclovir, Table 1). Slightly higher solubility increase was observed in PBS, where [Cho][Acy] was ca. 500 times more soluble than neutral acyclovir (660 mg active/mL vs. 1.33 mg active/mL for acyclovir, Table 1). In SIF, [Cho][Acy] was ca. 650 times more soluble than neutral acyclovir (649.1 mg active/mL vs. 0.95 mg active/mL for acyclovir, Table 1).

Even higher solubility for [Cho][Acy] was observed for the lower pH SGF buffer (861.8 mg acyclovir active/mL, Table 1). Interestingly, [Cho][Acy] was even ~6 times more soluble in water (600 mg active/mL) than the commercial form of the drug, acyclovir sodium (90.68 mg active/mL). Acyclovir in its cationic form (salt [H_2_Acy]Cl and API-IL [H_2_Acy][Doc]) was less buffer-soluble than acyclovir in its anionic form independent of the media chosen, however even compound [H_2_Acy]Cl was ca. 40–80 times more soluble than the neutral API itself.

## 8. Conclusions

In the pursuit of new antivirals, the pharma industry has made a lot of advances in drug discovery with the focus on the pharmacore. At the same time, new APIs in the form of ILs (API-ILs) from already known drugs can be a strategy to “repurpose” old drugs which can also be applied to drugs that were never commercialized for their originally developed goals. This approach results in “old new” pharmaceuticals that could potentially exhibit enhanced bioavailability, demonstrate modified physical attributes, expand delivery options, and prevent the common issues of drug resistance and reduced susceptibility. Yet, when presented with an option of using active ingredients as pure liquid salts, there is a lot of concern that indicates a lack of familiarity with this class of compounds and the absence of ready tools for their characterization, manufacture, and study, more than any real API-IL shortcomings.

With so much information amassed from API-IL research over the past 10 years, it is surprising that one still has to advocate for their potential. We have already shown that the poorly soluble antiviral drugs like acyclovir can be made into API-ILs taking advantage of its amphoteric character significantly enhancing its low systemic bioavailability as a result of ~650-fold improved aqueous solubility. It is encouraging, however, to see GSK’s recently reported reverse transcriptase inhibitor for the treatment of HIV virus in the form of an IL. GSK found that API-ILs significantly exceeded the maximum plasma drug concentrations which could be achieved by a typical solid suspension, but also expressed concern about the lack of GMP manufacturing equipment and tools to handle API-ILs.

We agree that there are many unanswered questions and challenges for API-IL implementation. The first impediment is addressing regulatory aspects necessary for eventual API-ILs to be offered. Regulations have to be developed to meet the challenge of these new promising liquid salts, and one of the questions here is how these API-ILs will be defined—as new APIs or drug product intermediates/in-process materials? The second obstacle is characterization. In the matter of confirming and establishing API-IL properties, existing protocols developed in academia could be a starting point, but there is a need of new methods of characterization (or at least methodologies) focused on these liquid salts. Lastly, one still needs to overcome multiple generated stereotypes and misconceptions about API-ILs, despite the fact that this is a class of compounds defined as being a salt that melts below 100 °C that could have almost unlimited variation in chemical composition.

Despite the risk of studying something new, we advocate that pharma really should consider the advantages that a particular API-IL could give. API-ILs could be the genesis of disruptive innovation once their complexity and unique attributes are more fully understood. This new awareness, new thinking will give one confidence to explore the transformational applications encapsulated in the IL form, as can be seen from the discussed examples.

## Figures and Tables

**Table 1:** The solubility of API-ILs and pharmaceutical salts of acyclovir in water and physiological buffers. Adapted from ref. [47].

Buffer or Water	mol/L	mg/mL	mg Active/ mL
**Acyclovir (3Acy·2H_2_O)**, mp = 256.5 °C (dec) ^b^			
**Water**	0.00699(1)	1.574(2)	1.495(2)
**PBS**	0.00631(3)	1.4(1)	1.33(1)
**SIF**	0.00444(1)	1.0(1)	0.95(1)
**Na[Acy] ^a^**, mp = 256 °C			
**Water**	0.404(5)	100.0	90.68
**[Cho][Acy] ^a^**, mp = ND ^c^			
**Water**	2.68(5)	880(20)	600(10)
**PBS**	2.92(5)	960(20)	660(10)
**SIF**	2.83(9)	930(30)	650(20)
**SGF**	3.8(1)	1300(500)	860(30)
**[P_4,4,4,4_][Acy] ^a^**, mp = 95.1 °C ^d^			
**Water**	1.66(3)	800(10)	373(7)
**[N_4,4,4,4_][Acy] ^a^**, mp = 70.6 °C			
**Water**	0.952(8)	444.7	213.7
**PBS**	0.9867(2)	490(30)	220(20)
**SIF**	0.6394(2)	300(20)	140(10)
**SGF**	0.7416(8)	350 (2)	170(20)
**[N_1,1,1,16_][Acy] ^a^**, mp = 48.3 °C ^e^			
**Water**	0.4496(25)	229(1)	101.3(5)
**[H_2_Acy]Cl ^a^**, mp >290 °C			
**Water**	0.241(1)	63.1(30)	54.4(3)
**PBS**	0.244(7)	64(2)	55(2)
**SIF**	0.339(7)	89(2)	76(2)
**SGF**	0.348(3)	91.0(8)	78.4(7)
**[H_2_Acy][Doc] ^a^**, mp = ND ^c^			
**Water**	0.01305(1)	8.461(5)	2.948(4)

^a^ Abbreviations used: acyclovir in anionic form, Na[Acy]—sodium acyclovir, [Cho][Acy]—choline acyclovir, [P_4,4,4,4_][Acy]—tetrabutylphosphonium acyclovir, [N_4,4,4,4_][Acy]—*N*,*N*,*N*,*N*-tetrabutylammonium acyclovir, [N_1,1,1,16_][Acy]—*N*-hexadecyl-*N*,*N*,*N*-trimethylammonium acyclovir. Acyclovir in cationic form, [H_2_Acy]Cl—acyclovir chloride, [H_2_Acy][Doc]—acyclovir docusate. ^b^ In agreement with reference [112]. ^c^ ND—not detected in the range from −60 to 120 °C. ^d^ Observed only during second and third DSC cycles. ^e^ Observed only during first DSC cycle.

## Abbreviations

U.S. FDA	U.S. Food and Drug Administration
[Cho][Acy]	Choline Acyclovir
[H_2_Acy][Doc]	Acyclovir Docusate
[H_2_Acy]Cl	Acyclovir Chloride
[N_1,1,1,16_][Acy]	*N*-hexadecyl-*N*,*N*,*N*-Trimethylammonium Acyclovir
[N_1,4,4,4_][Acy]	*N,N,N*-Tributyl-*N*-methylammonium Acyclovir
[N_4,4,4,4_][Acy]	*N*,*N*,*N*,*N*-Tetrabutylammonium Acyclovir
[P_4,4,4,4_][Acy]	Tetrabutylphosphonium Acyclovir
[P_4,4,4,4_][Ibu]	Tetrabutylphosphonium Ibuprofenate
API	Active Pharmaceutical Ingredients
API-IL	Active Pharmaceutical Ingredient—Ionic Liquid
BCS	Biopharmaceutical Classification System
COVID-19	Coronavirus Disease 2019
DLS	Dynamic Light Scattering
EUA	Emergency Use Authorization
FT-IR	Fourier-Transform Infrared
GMP	Good Manufacturing Practice
GSK	GlaxoSmithKline
HPLC	High-Performance Liquid Chromatography
HR-MAS	High-Resolution Magic Angle Spinning
IL	Ionic Liquid
NMR	Nuclear Magnetic Resonance
PBS	Phosphate Buffer Saline
PFG-NMR	Pulse-Field-Gradient
SGF	Simulated Gastric Fluid
SIF	Simulated Intestinal Fluid
